# *In silico* modelling of a cancer stem cell-targeting agent and its effects on tumour control during radiotherapy

**DOI:** 10.1038/srep32332

**Published:** 2016-08-30

**Authors:** Loredana G. Marcu, David Marcu

**Affiliations:** 1 Faculty of Science, University of Oradea, Oradea 410087, Romania; 2 School of Physical Sciences, The University of Adelaide, SA 5005, Australia

## Abstract

Head and neck cancers (HNC), like most solid tumours, contain a subpopulation of cancer stem cells (CSC) that are commonly responsible for treatment failure. Conventional therapies are unsuccessful in controlling CSCs, thus novel, targeting therapies are needed. A promising agent is ATRA (All-trans-retinoic acid) that was shown to induce CSC differentiation, cell cycle redistribution and CSCs radiosensitisation. To add to the limited data, this work simulated the effects of ATRA on a virtual HNC and evaluated tumour response to radiotherapy. A Monte Carlo technique was employed to grow a HNC consisting of all lineages of cancer cells. The biologically realistic input parameters led to a pre-treatment CSC population of 5.9%. The Linear Quadratic model was employed to simulate radiotherapy. ATRA-induced differentiation, cell arrest and apoptosis were modelled, based on literature data. While the effect of differentiation was marginal, the strongest influence on CSC subpopulation was displayed by ATRA’s cell arrest effect via an exponential behaviour of the dose-response curve. The apoptotic effect induced by ATRA shows linear correlation between the percentage of apoptotic cells and dose required to eradicate CSCs. In conclusion, ATRA is a potent CSC-targeting agent with viable impact on tumour control when combined with radiotherapy.

## Cancer Stem Cells (CSC) and their Role within the Tumour

Locally advanced head and neck carcinomas (HNC) are aggressive tumours due to several radiobiological factors such as: hypoxia, elevated levels of the endothelial growth factor receptor and accelerated repopulation during treatment. Beside these factors the response to irradiation is also dictated by tumour composition, mainly by the presence of stem-like cells that hold the ability to proliferate indefinitely.

The current literature is filled with an ever-growing body of evidence towards the existence of cancer stem cells. These cells represent a subpopulation of tumour cells that proliferate indefinitely, are tumorigenic and also more quiescent than non-cancer stem cells^1^. Furthermore, these cells are considered to be accountable for treatment resistance and failure, as well as tumour recurrence. The ability to divide symmetrically is another powerful tool that cancer stem cells use for their survival. Symmetrical division of cancer stem cells is the process whereby in mitosis, both resulting cells are stem cells. Since cancer growth is sustained by this small subpopulation of cancer stem cells, their symmetrical division further increases the subpopulation and, consequently, resistance to treatment.

Based on a study looking at the expression of CSC marker proteins in a head and neck squamous cell carcinoma xenograft mouse model, Geissler *et al.* have assumed that CSCs can be sub-classified into migratory and stationary cancer stem cells^2^. Consequently, stationary CSCs are quiescent in nature while migratory CSCs are active and invasive, thus being responsible for tumour growth and regrowth. The main properties of CSC are summarised below:

- Long-lived and have the ability to proliferate indefinitely (*Moore et al.*^1^)
- Can generate all heterogeneous lineages of the original tumour (*Al Hajj et al.*^3^)
- Can recreate themselves by symmetrical division *Morrison et al.*^4^
- Are more resistant than non-stem cancer cells *Moore et al.*^1^
- Preferentially reside in certain microenvironmental niches within the tumour (often prefer hypoxic environment) *Peitzsch et al.*^5^
- Can be classified into migratory CSCs (i.e. active and invasive) and stationary CSCs (i.e. quiescent, non-invasive) *Geissler et al.*^2^

Experimental studies on the phenotypic heterogeneity of tumours have shown that cancers comprise of various cell subpopulations with heterogeneous molecularity, which confers them distinct biological behaviour^2^. Consequently, among these varied subpopulations there are cell groups with different features that are linked to invasiveness and metastases, radioresistance, resistance to chemotherapy, etc. Eventually, the extent of the more aggressive subpopulations will dictate the fate of the tumour. This fact is prompting the need for the quantification of the CSC subpopulation.

Tumour cell line experiments have shown that the percentage of CSC varies severely among tumours of different histopathological type. While a low percentage of CSCs have been found in acute myeloid leukaemia (0.4%), colon cancers have been suggested to have a much higher subpopulation of CSCs (82.7%)^6^. In head and neck tumours, the first identification of CSC has been reported by Prince *et al.* who has isolated a cellular subgroup exhibiting stem-like properties^7^.

To date, the number of quantitative reports in the literature on the percentage of CSC in head and neck cancer is very scarce. The existing literature illustrate that there are significant differences among the studied head and neck cell lines. Accordingly, in the experiments conducted by Tang *et al.* the CSC proportion in various head and neck cell lines ranged between 1.7% and 13.5%^8^. The experiment undertaken by Harper *et al.* on HNC cell lines has indicated that the proportion of CSC in CaLH3 cell line is 12.3%^9^.

There are experimental studies that focused on the characterisation of CSC cells through stem cell markers. Given that the number of such studies is still scant and the results inconclusive, there is need for additional tools that could assist in explaining the impact of CSC subpopulations within head and neck cancers on treatment outcome^10^. It is therefore important to determine the pattern of CSC contribution to tumour proliferation during radiotherapy and its role in tumour recurrence by quantitative means.

## CSC and Resistance to Treatment

Advanced and unresectable head and neck cancers are traditionally treated with chemo-radiotherapy. Conventional radiotherapy delivers a total dose of 70Gy in 2Gy fractions over 7 weeks, with limited tumour control. However, tumour hypoxia and repopulation hinder the efficacy of conventional radiotherapy. A more effective schedule is hyperfractionated radiotherapy^11^, whereby smaller doses are delivered twice a day (1.2Gy per fraction) over the same time period, thus totaling 84Gy. This type of fractionation overcomes to certain extent tumour repopulation in between fractions and stimulates reoxygenation.

Cancer stem cells are an added challenge to head and neck radiotherapy as CSCs have been shown to be more resistant to radiation than differentiated or non-CSC^1^. This behaviour prompts for the development of CSC-targeted therapies that could sensitise the cells to the effect of radiotherapy.

## CSC-Targeting Therapies: All-Trans-Retinoic Acid (ATRA)

Given that the differentiated state assures better response to radiotherapy, a plausible strategy to increase tumour control would be to stimulate CSC differentiation. One of the agents that was found to exhibit a powerful differentiating potential is all-trans-retinoic acid (ATRA)^12^,^13^.

**Figure 1:**
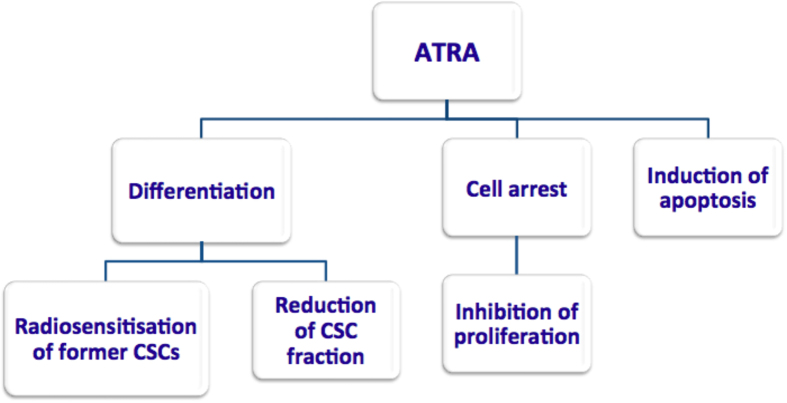
ATRA and its potential properties as indicated by *in vitro* and *in vivo* studies.

ATRA is a member of the retinoid family and is an active metabolite of vitamin A. Retinoids present a potent effect on cell growth, differentiation and apoptosis and influence multiple signaling pathways that are involved in stem cell preservation^14^. Both *in vitro* and *in vivo* pre-clinical studies showed that ATRA has powerful effects on CSCs by inducing cell cycle arrest due to the complexity of DNA damage and also apoptosis^12^,^13^. Furthermore, differentiation caused by ATRA was observed in the previously mentioned studies, which was associated with downregulation of the Wnt pathway, a central mechanism controlling malignant transformation. The overall effects of ATRA have led to a decrease in the surviving fraction when ATRA was combined with radiotherapy^13^ (Fig. 1).

CSC-targeting agents, including ATRA, represent an innovative approach towards cancer management and they are currently being investigated in pre-clinical settings. The present work is therefore an added tool that allows the investigation of the potential effects of ATRA on head and neck cancer.

## The Aim and Justification of the Current Work

The aim of this work is to integrate the properties of CSCs into a virtual hypoxic head and neck cancer grown via Monte Carlo techniques in order to quantify the behaviour of CSC during hyperfractionated radiotherapy. Another important goal was to evaluate tumour response as a function of the interplay between radiotherapy and ATRA.

The current work focuses on head and neck squamous cell carcinomas, as they are one of the most radiobiologically challenging tumours. The reason to choose a stochastic approach to model tumour growth and response to treatment is to better fit the biological reality whereby the cellular phenotype, malignant growth, cellular damage, and cell kill due to either natural causes or irradiation, are all driven by probabilistic phenomena.

Furthermore, within the model, each cell is followed throughout its life and treatment, knowing its age, phenotype, position in the cell cycle, number of generations and its oxygenation level. Due to the lack of quantitative data regarding the CSC division pattern within head and neck tumours, the goals of the current work are:

1. To quantify the fraction of CSC during hyperfractionated radiotherapy and evaluate the influence of symmetrical division of CSCs on tumour response to treatment;
2. To implement the properties of ATRA (i.e. differentiation, cell arrest, apoptosis) in the model and to evaluate the efficacy of each property on tumour response during radiotherapy;
3. To analyse the effect of the CSC-targeting agent on the tumour as a whole.

## Results

All the results shown below have been achieved by treating the virtual tumour with hyperfractionated radiotherapy. The first section shows the effect of high symmetrical division probabilities on tumour response to radiotherapy alone, while all other sections present the results of ATRA when combined with hyperfractionated radiotherapy.

### The influence of symmetrical division on CSC subpopulation and response to hyperfractionated radiotherapy

**Figure 2:**
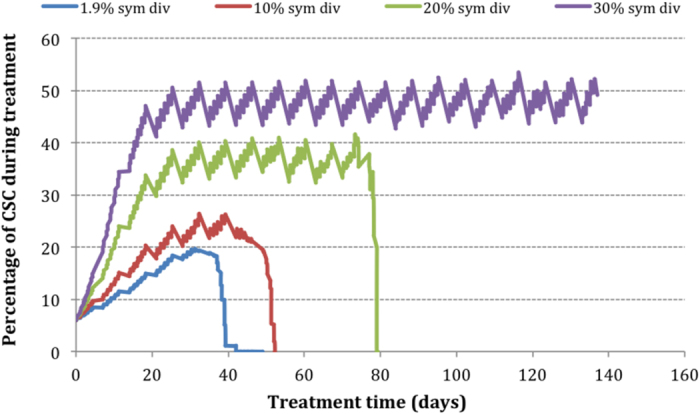
The impact of symmetrical division probability on CSC subpopulation in a moderately hypoxic HNC.

To illustrate the impact of symmetrical division probability (SDP) on CSC subpopulation a scenario involving moderately hypoxic HNC with various SDPs for cancer stem cells has been modeled (Fig. 2). SDP has been varied from the initial 1.9% up to a percentage that resulted in uncontrolled tumour growth, which was found to be 30%. The aim was to determine the treatment time required for complete tumour eradication and to evaluate the quantitative link between the probability of symmetrical division and the percentage of CSCs in the tumour.

Tumour eradication has not been achieved within the clinically allocated treatment time (7 weeks) for percentages of CSC SDP larger than (or equal to) 10%. For percentages as high as 30 and over, the tumour seems uncontrollable, at least with the current treatment technique. Comparing the plateau values in Fig. 2, it is observed that each 10% increase in the probability of symmetrical division leads to 10% increase in the percentage of CSC subpopulation (particularly for tumours with a SDP greater than 10%).

### The effects of ATRA on cancer stem cells

**Figure 3:**
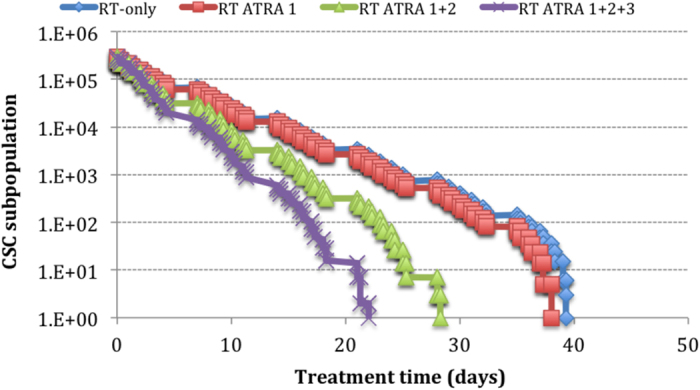
Survival curves of the CSC subpopulations under hyperfractionated radiotherapy and various ATRA effects for 0.1% SDP.

Three main properties of ATRA have been modelled: differentiation (ATRA 1), cell cycle arrest (ATRA 2), and induction of apoptosis (ATRA 3). The differentiation mechanism manifests through the loss of symmetrical division of stem cells. Both the independent as well as the combined effect (ATRA1+2+3) of the above properties have been studied (Fig. 3).

Since differentiation yields in reduction of SDP, the effect of this mechanism can be deducted from Fig. 2. For a tumour that exhibits SDP of 20%, if differentiation reduces this value to 10%, Fig. 2 shows that the ATRA-induced effect results in a dose de-escalation of 32%. Applying the same principle to the tumour with 30% SDP presented in Fig. 2, we observe that a drop of 10% due to ATRA differentiation would control a tumour that otherwise was uncontrollable.

**Figure 4:**
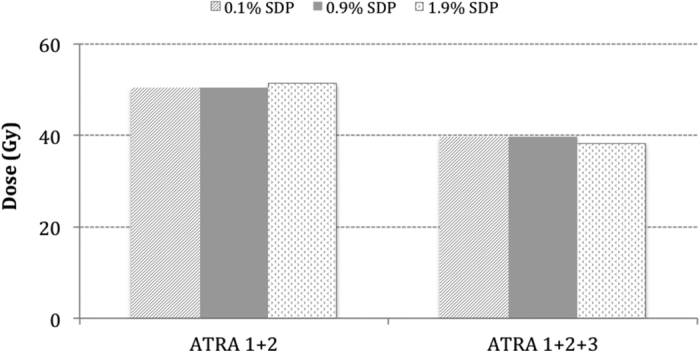
The effect of differentiation when combined with cell arrest without apoptosis (ATRA 1+2) and with apoptosis (ATRA 1+2+3).

The head and neck tumour studied in the employed model displays a naturally low SDP of 1.9%, which negates the effect of ATRA-induced differentiation in this particular tumour. Therefore, as shown in Figs 3 and 4, there is no difference in CSC response when the SDP is varied from 1.9% to 0.1%. The surviving curves in Fig. 3 that represent the RT-only scenario and RT+ATRA1 show minimal difference (3 dose fractions).

Figure 4 illustrates the effect of differentiation when combined with cell arrest with or without apoptosis. The differences in the total dose to control the CSC subpopulation among tumours with varied SDP are 1.1 Gy with cell arrest and 1.2 Gy with cell arrest and apoptosis.

The effect of differentiation through reduction of SDP was marginal due to the already small pre-treatment SDP of 1.9%. This parameter would have a higher impact in tumours with large initial SDPs, similar to the hypothetical scenarios presented in Fig. 3. Due to this result all further simulations have been processed with 1.9% SDP as in the initial tumour growth model.

However, it is to be noted that by simply arresting the cells in the G_2_ phase, ATRA inhibits proliferation thus inducing a significant improvement in CSC response to hyperfractionated radiotherapy. With cell arrest (ATRA1+2), there is a 12-fraction difference as compared to radiotherapy-alone for the same tumour response. This corresponds to a dose de-escalation of 14.4 Gy. Also, the effect of apoptosis decreases the total dose needed for CSC eradication with a further 10.8 Gy.

### The effect of ATRA on cell cycle distribution

To investigate the dose-dependent response of CSC to ATRA, various scenarios involving different percentages of CSCs arrested in the G2 phase have been simulated (as described in Methods) for a fixed percentage of apoptotic death among the blocked cells. Extreme percentages (10% and 90%) have been modelled to evaluate the least optimal and the best-case scenarios.

**Figure 5:**
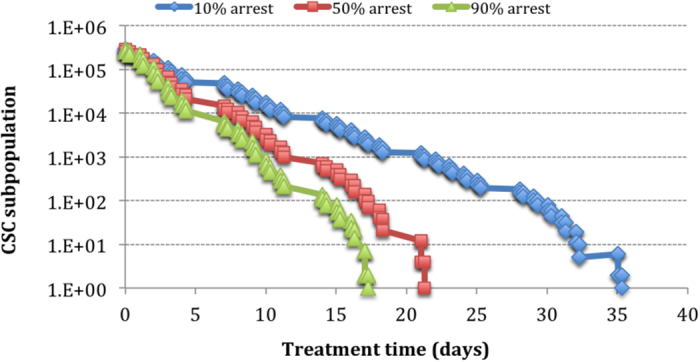
The effect of cell arrest induced by ATRA on the CSC subpopulation.

Figure 5 shows a supra-linear relationship between the fraction of cell cycle arrest and CSC subpopulation response to treatment, thus implying a very potent effect on the overall response.

### ATRA and induction of apoptosis

Similarly to the effect of cell arrest, apoptosis was simulated within the (10–90%) range as described in Methods, for a fixed percentage of cells arrested in G_2_.

**Figure 6:**
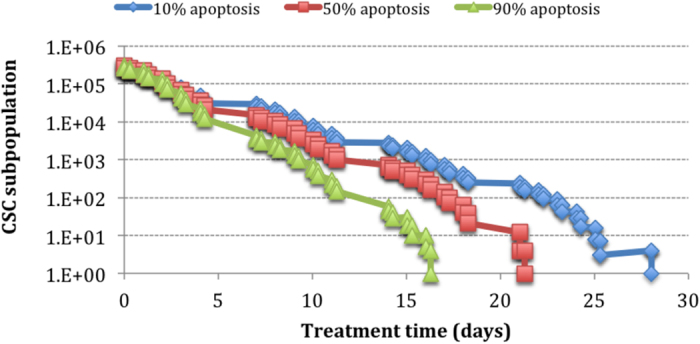
The effect of apoptotic death caused by ATRA on the CSC subpopulation.

The effect of apoptosis on CSC response seems to increase linearly with the percentage of apoptotic cells (Fig. 6), thus showing a less pronounced influence as opposed to the effect of cell arrest.

### Comparison of ATRA effects on CSCs

**Figure 7:**
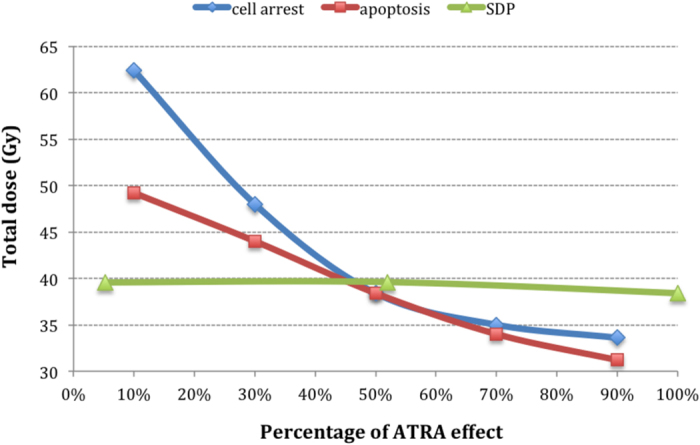
Comparison of the ATRA effects on CSCs.

The individual effects of ATRA on CSCs have been presented in one graph to illustrate their influence on tumour response. The current parameters for the modelled HNC allow little variation for SDP. Given the initial low percentage of 1.9%, any further reduction has a negligible effect on tumour control, suggested by the constant line in Fig. 7. To enable the representation of the three effects in one common graph, the initial SDP of 1.9% has been considered 100% of possible effect.

The apoptotic effect induced by ATRA shows linear correlation between the percentage of apoptotic cells and dose required to eradicate CSCs.

Unexpectedly, the strongest influence on CSC subpopulation control has been displayed by ATRA’s cell arrest effect, illustrated by an exponential behaviour of the dose-response curve (Fig. 7).

## Discussion

The current work has analysed the radiobiological challenges raised by the CSC subpopulation in a virtual hypoxic head and neck cancer. The model has simulated the properties of cancer stem cells during radiotherapy and has shown that the percentage of CSCs increases considerably during treatment, due to their radioresistance. This result is in accordance with the literature findings^13^.

The ability to divide symmetrically is a potent tool of CSCs, and yet to be quantified. Due to the lack of such data in the scientific literature, the model has considered various scenarios when the symmetrical division probability is increased during radiotherapy and has concluded that high percentages of symmetrical division of CSCs enable uncontrolled tumour proliferation. Quantification of symmetrical division probability shows the high impact of this parameter on tumour composition. Tumour eradication has not been achieved within the clinically allocated treatment time (7 weeks) for percentages of CSC SDP larger than (or equal to) 10%.

Thus high percentages of SDP represent a challenge in radiotherapy, reason why CSC-targeting agents are currently trialed in pre-clinical studies. All-trans-retinoic acid has proven to be an effective agent with a potential to induce cell arrest and apoptosis.

To add to the literature data, we have developed an *in silico* tool to study the three main features of ATRA: differentiation, cell cycle arrest, and induction of apoptosis.

The effect of differentiation through reduction of SDP was minimal, which is due to the already small pre-treatment SDP of 1.9%. This parameter would have a higher impact in tumours with large SDPs, similar to the hypothetical scenarios presented in Fig. 2.

Induction of cell arrest by ATRA is a potent targeting approach in HNC. The G_2_ phase of the cell cycle is known to have an increased radiosensitivity, therefore cells that are arrested in this phase in order to repair their ATRA-induced DNA damage, are also more exposed to cell kill during radiotherapy. Furthermore, given that HNC are rapidly growing tumours, meaning that the cycling population exhibits greater kinetics along the cell cycle, the two doses of radiation administered during hyperfractionated radiotherapy are an added benefit to efficient tumour kill. When employing various percentages of cell arrest, the model showed an exponential correlation between this parameter and the tumoricidal dose.

Apoptotic cell death induced by ATRA was shown to be linear in relation to the total dose. For both cell cycle arrest and apoptosis, extreme values have been simulated in order to analyse the least optimal as well as the best-case scenarios.

Given the low initial rate of symmetrical division, which denied ATRA the possibility to intervene as a differentiating agent, ATRA was proven to be a potent radiosensitising and CSC-targeting agent for head and neck cancer with viable impact on tumour control when combined with radiotherapy. This shows the critical importance of determining quantitative values for the pre-treatment CSC subpopulation and for the rate of symmetrical division.

Each histopathological tumour type is likely to have its own value for the above-mentioned parameters and we expect the cell arrest and apoptotic effects to remain exponential and linear, respectively, with differentiation adding another strong influence on treatment outcome.

Therefore, given the dose-dependent response of CSCs to ATRA, it is crucial to obtain both quantitative and qualitative knowledge on CSC dynamics in order to describe the resistant subpopulation and to design efficient and less toxic treatment regimens in head and neck oncology.

## Methods

### Simulation of head and neck tumour growth

The first step of this work was the growth simulation of a head and neck cancer, having biologically realistic growth kinetic parameters. Consequently, the head and neck cancer grown within the model has some standard values, in good correlation with the literature averages as shown in Table 1.

The tumour growth algorithm follows a Monte Carlo technique that takes probabilistic decisions based on input parameter values obtained from the literature (Table 1). The tumour growth module has been described previously^15^,^16^. The tumour growth module generates a probabilistic value for the type of new cell to be created. The hierarchical cell lineage is comprised of (1) cancer stem cells (CSC), (2) differentiated cells (D) and (3) quiescent or resting cells (Q). Cancer stem cells have the ability to procreate indefinitely and can undergo symmetrical division by giving birth to two stem cells, while differentiated cells are only able to contribute to tumour growth for 3 generations after which they die. Quiescent cells reside in the G_0_ phase outside the cell cycle and represent about 85% of the total cell population, value that has been obtained through multiple iterations. With every cell generation, a new cell-cycle time is allocated to both the original as well as the newly created cell. These cell-cycle time values are generated according to an asymmetrical Gaussian distribution truncated at one standard deviation towards lower values and 2 standard deviations towards higher values. This approach is in agreement with the literature data whereby the average head and neck cell cycle time is 33 h, ranging from 20 h to 60 h^17^. The tumour has been grown until a clinically detectable size of 10^7^ cells.

The CSC subpopulation modelled here has biological and radiobiological characteristics according to the latest experimental findings. Consequently, CSCs in the current model exhibit: indefinite proliferation capacity^1^, CSC-specific division pattern (both symmetrical and asymmetrical division)^3^ and the ability to generate all heterogeneous lineages of the original tumour^3^. Furthermore, the radioresistance of CSCs was established according to the literature^18^ and adapted for HNC. Since hypoxia is an important factor that contributes to the overall tumour response to radiotherapy, the virtual head and neck cancer is considered to be moderately hypoxic, in order to represent the average patient group.

Every 24 hours the model generates a set of statistical results based on its current state that are stored in a result file for later interpretation. All random generators employed by the model are using the Mersenne Twister algorithm (a pseudorandom number generator) in its 32 bit implementation, allowing for generation of good quality random numbers at sensible speed.

### Modelling radiotherapy with ATRA as CSC-targeting agent

Proliferating cells cycle along the four phases of the cell cycle (M, G_1_, S, G_2_) whereas non-proliferating cells rest in the quiescent phase (G_0_). Each phase of the cycle has been correlated with a phase-specific surviving fraction derived from the literature^19^. Surviving fractions have been determined using the Linear Quadratic model starting from the premise that the average surviving fraction of HNC after 2Gy is 54%^17^.

The effects of ATRA, as a CSC-targeting agent have been implemented into the model. The three effects that have been suggested by the literature to be of importance when targeting CSCs are: differentiation, cell cycle arrest and apoptosis^12^,^13^.

Differentiation has been simulated by reducing the symmetrical division probability from the initial 1.9% (see Table 1) to 0.1% in a step-like manner. Cell cycle arrest in the G_2_ phase has been modelled based on the work of Bertrand *et al.*^13^. They have shown that administration of ATRA prior to radiotherapy has increased the CSC population in the G_2_/M phase for up to 48 h, with a peak of 80% cells being arrested after 24 h following radiotherapy. Studies in mice have shown that the effect of ATRA on CSCs is dose-dependent^12^. In the model we have considered the effects of various doses of ATRA leading to different percentages of cells being arrested (from 10% to 90% cell arrest). Cells are blocked in the G_2_ phase in order to undergo radiation-caused DNA damage repair. However, part of the cells will not be able to repair their DNA breaks, reason why they will die via apoptosis. Regarding the percentage of cells that are eliminated through apoptosis, a sensitivity study has been conducted with values from 10 to 90%. To sum up, the three variables that have been modelled as the effect of ATRA on CSCs are as follows:

- Differentiation, by varying the symmetrical division probability (0.1–1.9%);
- Cell arrest, by varying the percentage of cells blocked in G_2_ (10–90%).
- Apoptosis, by varying the percentage of cells arrested in G_2_ that undergo apoptosis (10–90%).

## Additional Information

**How to cite this article**: Marcu, L. G. and Marcu, D. *In silico* modelling of a cancer stem cell-targeting agent and its effects on tumour control during radiotherapy. *Sci. Rep.* **6**, 32332; doi: 10.1038/srep32332 (2016).

## Figures and Tables

**Table 1:** Tumour growth parameters.

Tumour growth parameters	Model values
Input parameters	
Length of S phase	11 h^17^
Mean cell cycle time (range)	33 h (20 – 60h)^19^
Duration (proportions) of cell cycle phases	M:7%; G_1_:40%; S:30%; G_2_:23%^17^
Cell loss factor	85%^19^
Model-derived parameters	
Volume doubling time	52 d^20^
Labelling index	4.7%^21^
Cell division rate (24h)	1.3%
Pre-treatment probability of CSC symmetrical division	1.9%
Pre-treatment percentage of CSCs	5.42%
